# Artificial intelligence in hepatology: A comprehensive scoping review of clinical applications, challenges, and future directions

**DOI:** 10.1016/j.iliver.2025.100205

**Published:** 2025-11-07

**Authors:** Kirolos Eskandar

**Affiliations:** Faculty of Medicine and Surgery, Helwan University, Cairo 12511, Egypt

**Keywords:** Digital pathology, Clinical decision support, Predictive analytics, Medical imaging algorithms, Liver transplantation outcomes, Scoping review

## Abstract

**Background and aims:**

Artificial intelligence (AI) is increasingly integrated into hepatology, yet the existing evidence is fragmented. This scoping review systematically mapped clinical AI applications across hepatology, summarized validated outcomes, and highlighted implementation challenges and research priorities.

**Methods:**

Following the Arksey–O’Malley and Levac frameworks and reported in accordance with PRISMA-ScR, a systematic search of PubMed, Embase, Scopus, Web of Science, and IEEE Xplore (January 2018–July 2025), complemented by grey-literature screening, identified relevant studies. Two reviewers independently screened and extracted data. From 3214 records, 75 studies met inclusion criteria. (PROSPERO ID: CRD420251159117).

**Results:**

Most studies were retrospective and single-center. Imaging models achieved area-under-curve values of 0.80–0.95 for fibrosis staging, lesion detection, and volumetry, often comparable with expert radiologists. Digital-pathology algorithms enabled objective quantification of fibrosis and steatosis. Machine-learning tools improved prediction of disease progression, readmission, and mortality compared with conventional scores, while AI-based transplantation models enhanced donor–recipient matching and graft-survival forecasting. Natural-language-processing systems facilitated early complication detection from electronic health records. Common barriers included small datasets, limited external validation, and model interpretability concerns.

**Conclusions:**

AI demonstrates strong potential for diagnostic, prognostic, and workflow enhancement in hepatology. Its responsible translation requires multimodal data integration, explainable modeling, prospective and multicenter validation, and equity-focused deployment strategies.

## 1 Introduction

Artificial intelligence has rapidly become a transformative force in medicine, offering new approaches for disease detection, prognostication, and treatment decision-making. Advances in machine learning (ML), deep learning (DL), natural language processing (NLP), and big data analytics have enabled extraction of clinically relevant insights from complex datasets such as imaging, histopathology, laboratory results, and electronic health records (EHRs). Applications in radiology, pathology, and public health have already demonstrated improved accuracy, speed, and consistency, with several AI-enabled systems approved for clinical use.^1^^,^^2^

Liver disease represents a major global health burden, encompassing metabolic-associated steatotic liver disease (MASLD, formerly NAFLD), viral hepatitis, cirrhosis, hepatocellular carcinoma (HCC), and cholangiopathies. Rising prevalence—driven by obesity, diabetes, and aging populations—has created substantial diagnostic and prognostic challenges. Current pathways often depend on invasive, expensive, or inconsistent diagnostic tools, while prognostic stratification remains difficult due to disease heterogeneity and comorbidities.^3^ AI offers potential solutions by enabling noninvasive fibrosis staging, early detection of malignant transformation, prediction of decompensation, donor–recipient matching optimization in transplantation, and integration of multimodal data for precision care. Emerging studies, including regional research from the Middle East and North Africa, demonstrate optimism toward AI adoption but also reveal gaps in infrastructure, clinician training, and real-world readiness.^4^

Despite rapid progress, significant challenges persist. Many AI models remain retrospective, single-center, and limited in external validity. Issues of interpretability, algorithmic bias, and regulatory inconsistency continue to hinder clinical translation. These factors underscore the need for a structured overview of how AI is currently applied in hepatology and where evidence gaps remain.^5^^,^^6^

This scoping review uniquely maps clinical AI applications across imaging, histopathology, chronic liver disease, cirrhosis, and transplantation and synthesizes validation practices and equity reporting between January 2018 and July 2025. By systematically charting this landscape, the review highlights current clinical implementations, identifies methodological limitations, and proposes research priorities for safe, equitable, and evidence-based integration of AI into hepatology practice.

## 2 Methodology

This scoping review was conducted in accordance with the methodological framework proposed by Arksey and O'Malley (2005), refined by Levac et al. (2010), and updated by the Joanna Briggs Institute (JBI) guidance.^7^, ^8^, ^9^ Reporting followed the Preferred Reporting Items for Systematic Reviews and Meta-Analyses extension for Scoping Reviews (PRISMA-ScR) to ensure transparency and rigor.^9^ The review protocol was registered with the International Prospective Register of Systematic Reviews (PROSPERO; registration ID: CRD420251159117).

### 2.1 Search strategy

A comprehensive search was conducted in PubMed/MEDLINE, Embase, Scopus, Web of Science, and IEEE Xplore to identify studies published between 1 January 2018 and 21 July 2025. In total, 3214 records were identified across all databases before deduplication. Earlier versions of the search summary reported per-database subsets; the full count is now presented for consistency with PRISMA-ScR standards. Search strategies combined controlled vocabulary (e.g., MeSH, Emtree) and free-text terms for:

(1) Artificial intelligence (e.g., “artificial intelligence,” “machine learning,” “deep learning,” “neural networks,” “natural language processing”),
(2) Hepatology and liver disease (e.g., “hepatology,” “liver disease,” “cirrhosis,” “hepatocellular carcinoma,” “metabolic-associated steatotic liver disease,” MASLD, NAFLD, “NASH,” “hepatitis”), and
(3) Clinical applications (e.g., “diagnosis,” “prediction,” “risk stratification,” “transplantation,” “prognosis”).

Because some relevant digital-pathology and earlier automated-analysis studies do not explicitly use the labels “artificial intelligence” or “machine learning” in titles or abstracts, the final search strings (Supplementary Table S1) were expanded to include additional synonymous terms such as “assessment,” “automated analysis,” “computer-assisted diagnosis,” “digital pathology,” “qFibrosis,” and related phrases. These terms were verified retrospectively and did not identify any additional eligible studies beyond those already included.

The full Boolean search strings for each database are provided in Supplementary Table S1, with the last search performed on 21 July 2025. To minimize publication bias, reference lists of included studies and relevant reviews were screened, along with grey-literature sources such as conference abstracts, organizational reports (e.g., WHO, AASLD, EASL), and clinical-trial registries (ClinicalTrials.gov and WHO ICTRP).

### 2.2 Eligibility criteria

#### 2.2.1 Inclusion criteria

(1) Population: Human participants with any liver-disease diagnosis, including viral hepatitis, metabolic-associated steatotic liver disease (MASLD, formerly NAFLD), cirrhosis, hepatocellular carcinoma (HCC), cholangiopathies, and liver-transplant recipients.
(2) Intervention: Use of AI, ML, DL, NLP, or hybrid approaches for hepatology-related tasks (diagnosis, prognosis, treatment prediction, imaging, histopathology, transplantation, or workflow optimization).
(3) Outcomes: Clinical-performance metrics (e.g., accuracy, sensitivity, specificity, Area Under the ROC Curve (AUROC)), prognostic outcomes, treatment response, or workflow/operational metrics.
(4) Study design: Original research (retrospective, prospective, or mixed-methods) and evidence syntheses if they provided unique data.
(5) Language/publication status: English, peer-reviewed. Preprints were excluded unless subsequently published in peer-reviewed journals by July 2025.

#### 2.2.2 Exclusion criteria

(1) Preclinical, in silico, or purely technical studies without clinical relevance.
(2) Editorials, commentaries, letters, or opinion pieces lacking primary data.
(3) Articles unrelated to hepatology or clinical liver-disease practice.
(4) Duplicate or overlapping datasets describing the same clinical cohort were consolidated to the most complete publication.

### 2.3 Study selection

All records were imported into EndNote (Clarivate Analytics) for deduplication. Screening of titles, abstracts, and full texts was performed independently by two reviewers; disagreements were resolved by discussion until consensus was reached.

**Fig. 1:**
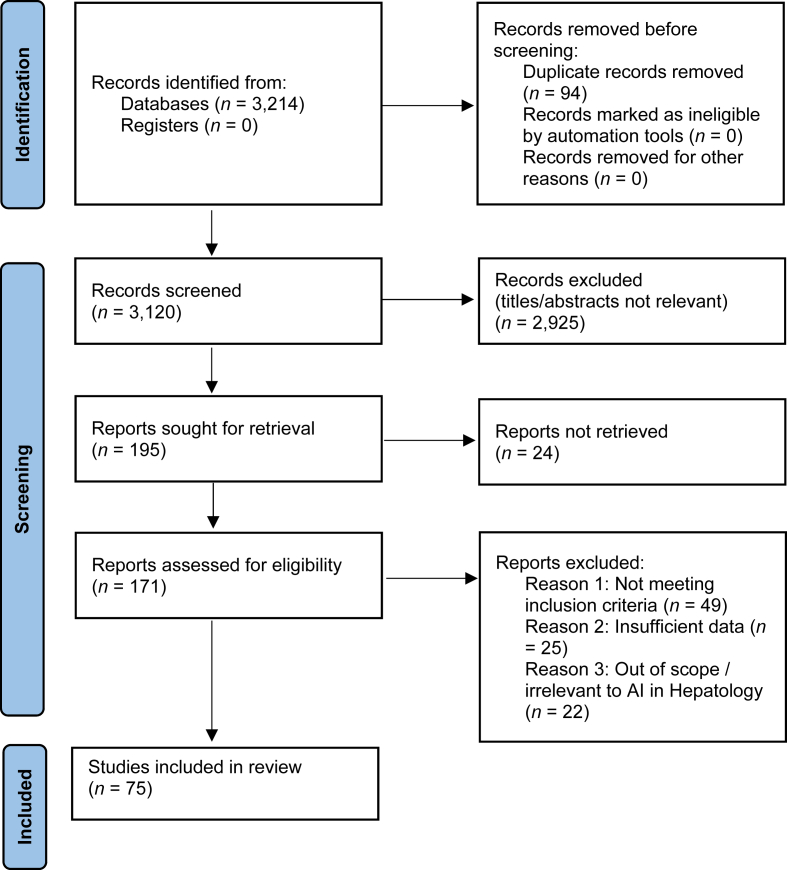
PRISMA flow diagram.

Records were excluded at the full-text stage if they did not meet inclusion criteria—specifically, studies that were (1) not clinical or human, (2) purely technical developments without clinical validation, (3) duplicate patient cohorts already represented elsewhere, or (4) lacking sufficient outcome data. The study-selection process is summarized in the PRISMA flow diagram (Fig. 1).

In total, 3214 records were identified across all databases. After removal of 94 duplicates, 3120 records were screened at the title and abstract level, with 2925 excluded as not relevant. 171 full-text articles were assessed for eligibility, and 95 were excluded (49 not meeting inclusion criteria, 25 with insufficient data, and 22 out of scope). Seventy-five studies were included in the final synthesis.

### 2.4 Data charting and extraction

Data from the 75 included studies were charted using a standardized extraction form (provided in Supplementary Table S2) that had been piloted on five studies to ensure consistency.

Extracted variables included:

(1) Bibliographic details (author, year, country, journal),
(2) Study design and data source characteristics (sample size, single vs multicenter, retrospective vs prospective),
(3) AI approach (ML, DL, NLP, hybrid),
(4) Clinical application (imaging, histopathology, chronic-liver-disease management, cirrhosis and complications, transplantation, workflow optimization),
(5) Outcomes reported (diagnostic accuracy, prognostic performance, clinical or operational utility),
(6) Validation approach (internal, external, prospective),
(7) Key findings, limitations, and authors' recommendations.

Extraction was conducted independently by two reviewers, with discrepancies resolved by consensus.

### 2.5 Data synthesis

Findings were synthesized narratively, grouped by clinical domain (imaging, histopathology, chronic liver disease, cirrhosis and complications, transplantation, and workflow optimization). Thematic synthesis highlighted methodological approaches, reported outcomes, and recurring challenges. Descriptive tables and figures (evidence maps, timelines) summarize study characteristics and trends.

### 2.6 Quality appraisal

Consistent with JBI guidance for scoping reviews, no formal risk-of-bias assessment was undertaken, as the goal was to map the breadth of available evidence rather than evaluate intervention effectiveness.^9^

### 2.7 Ethical considerations

This review analyzed previously published literature and therefore did not require ethical approval.

## 3 Overview of artificial intelligence in medicine

AI in medicine encompasses computational approaches that learn patterns from clinical data for diagnostic, prognostic, or workflow applications.^10^ Modern practice spans ML models that analyze structured data and DL architectures that extract features directly from imaging, pathology, and text. These methods have shown utility across hepatology tasks such as fibrosis staging, lesion detection, and digital pathology quantification. Key performance metrics include discrimination (AUC, sensitivity, specificity), calibration, and external validation—essential for assessing generalizability. A concise summary of core AI methods and their clinical relevance is provided in Supplementary Table S3.

## 4 Results

**Fig. 2:**
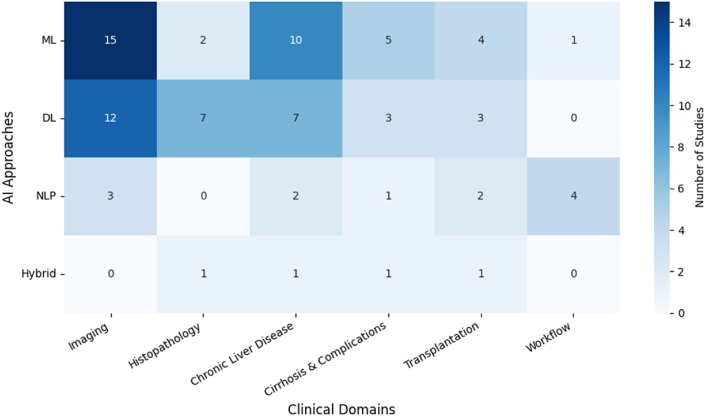
**Evidence map of artificial intelligence applications in hepatology.** Distribution of the 75 included studies across major clinical domains (columns) and AI approaches (rows). Imaging and chronic liver disease were the most frequently studied domains, predominantly using ML and DL. Fewer studies applied NLP or hybrid approaches, with limited exploration in workflow optimization. Values represent the number of studies per category. **Abbreviations:** AUC = area under the ROC curve; CNN = convolutional neural network; DL = deep learning; ML = machine learning; CPA = collagen proportionate area; MASLD = metabolic-associated steatotic liver disease; NAFLD = nonalcoholic fatty liver disease; NASH = nonalcoholic steatohepatitis.

Seventy-five studies met the inclusion criteria, spanning diverse clinical domains including imaging (*n* = 30), histopathology (*n* = 10), chronic liver disease (*n* = 20), cirrhosis and its complications (*n* = 10), transplantation (*n* = 10), and workflow optimization (*n* = 5). Several studies addressed more than one domain (for example, imaging applied to cirrhosis prognosis or transplantation planning), accounting for the apparent overlap between category totals. A concise overview of study distribution across domains and AI approaches is provided in Fig. 2. Across the 171 full-text articles screened, 96 were excluded, comprising 49 that did not meet the inclusion criteria, 25 with insufficient data, and 22 lacking direct clinical relevance to AI in hepatology. A detailed summary of excluded reports and reasons is presented in Supplementary Table S2.

To ensure completeness in the digital pathology domain, an expanded targeted search using the additional synonyms (“automated analysis,” “computer-assisted diagnosis,” “digital pathology,” “qFibrosis,” “collagen proportionate area”) was retrospectively verified. This confirmed that all eligible digital pathology studies (e.g., Gawrieh 2020; Ratziu 2024; Komura 2024; Grignaffini 2024) had been captured in the original synthesis, while digital-only reports lacking clinical validation were documented during screening but ultimately excluded as non-clinical.

### 4.1 AI in liver imaging

AI has rapidly transformed liver imaging by enabling automated detection, characterization, and quantification of hepatic pathology across CT, MRI, and ultrasound (US). DL–based models in particular have shown strong performance for lesion classification and staging. For example, a CNN applied to multiphase CT achieved 0.84 accuracy and an AUC of 0.92 for lesion categorization.^11^ In MRI, a CNN ensemble trained on 1210 patients (31,608 images) reported near-radiologist performance for multi-class tumor classification, with peak internal AUCs up to 0.98 depending on contrast phase.^12^ In ultrasound, a YOLOv5-based system trained on 26,288 images from 5444 patients achieved a focal-liver-lesion detection rate of 84.8% and malignant–benign discrimination with sensitivity and specificity of ∼97%, and per-lesion HCC detection of ∼82%.^13^ Collectively, these representative examples illustrate DL models achieving near-expert performance for focused classification or detection tasks across modalities.

**Fig. 3:**
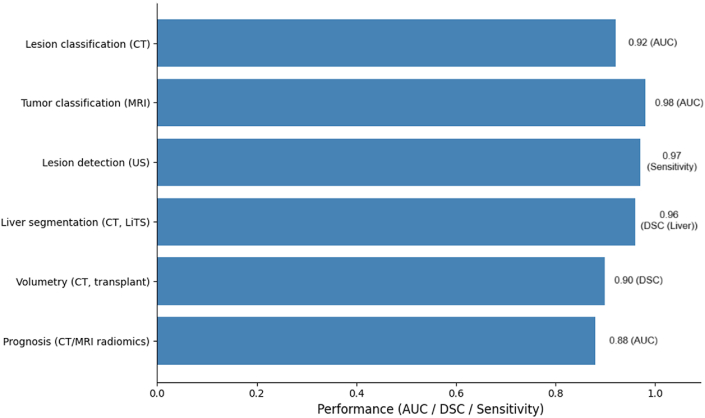
**Performance of artificial intelligence models in liver imaging.** Summary of reported diagnostic and prognostic performance across imaging modalities and tasks. Convolutional neural networks achieved high accuracy for lesion classification on CT (AUC 0.92) and tumor classification on MRI (AUC 0.98). Ultrasound models demonstrated strong lesion detection with sensitivity 0.97. Automated liver segmentation in the LiTS challenge achieved Dice similarity coefficients (DSC) up to 0.96 for whole-liver contours, while transplant volumetry reached DSC 0.899 with volumetric correlation R^2^ 0.996. Radiomics-based models provided prognostic value, with meta-analyses reporting pooled AUC 0.88 for recurrence prediction. **Abbreviations:** AUC = area under the ROC curve; CNN = convolutional neural network; CT = computed tomography; DSC = Dice similarity coefficient; DL = deep learning; ML = machine learning; MRI = magnetic resonance imaging; US = ultrasound; LiTS = Liver Tumor Segmentation challenge. **Note:** Reported metrics reflect internal or benchmark validations unless otherwise specified.

Automated segmentation and volumetry represent another mature application area. Multi-institutional benchmarks such as the Liver Tumor Segmentation (LiTS) challenge have driven algorithmic progress, with state-of-the-art methods achieving whole-liver Dice similarity coefficients (DSC) in the high 0.80 s to mid 0.90 s (best = 0.96), while tumor segmentation remains more challenging (DSC ∼0.67–0.74).^14^^,^^15^ In a living-donor CT cohort (*n* = 191), a 3D attention–CLSTM U-Net achieved a liver DSC of 0.899 and excellent volumetric agreement with manual volumes (R^2^ up to 0.996 for right-lobe volumetry), supporting its use in transplant planning^16^ (Fig. 3). However, lesion-level recall remains limited (<0.60 in LiTS analyses), highlighting persistent challenges for small-lesion detection.

Radiomics and AI-driven predictive modeling extend imaging utility beyond detection to prognosis and treatment response. A meta-analysis of radiomics in hepatocellular carcinoma (HCC) (10 studies; *n* = 1929) demonstrated pooled sensitivity 0.79, specificity 0.83, and AUC = 0.88 for early recurrence prediction after hepatectomy, outperforming clinical models.^17^^,^^18^ Radiomics-clinical nomograms across multiple cohorts have reported validation AUCs of 0.75–0.90 for recurrence prediction, microvascular invasion inference, and treatment response, with MRI peritumoral radiomics reaching AUCs = 0.82–0.85 in external validation.^19^^,^^20^ While performance varies by imaging phase and feature selection, these results collectively demonstrate the potential of AI to augment radiologic interpretation and prognostic modeling (Table 1).

**Table 1:** Selected applications of AI in liver imaging.

Task	Modality	Sample size	AI method	Validation	Key performance
Lesion classification	CT (multiphase)	*n* = 100+	CNN	Internal	Accuracy 0.84; AUC 0.92^11^
Tumor classification	MRI	*n* = 1210 (31,608 images)	CNN ensemble	Internal	AUC up to 0.98^12^
Lesion detection	Ultrasound	*n* = 5444 (26,288 images)	YOLOv5	Internal	Sens 97%; Spec 97%; per-lesion HCC detection ∼82%^13^
Liver segmentation	CT (LiTS challenge)	Multi-institution	U-Net variants	Benchmark	DSC liver 0.96; tumor 0.67–0.74^14^^,^^15^
Volumetry (transplant)	CT (living donor)	*n* = 191	3D attention–CLSTM U-Net	Internal	Liver DSC 0.899; R^2^ = 0.996^16^
Prognosis (recurrence)	CT/MRI radiomics	*n* = 1929 (meta-analysis)	Radiomics + ML	Meta-analysis	Sens 0.79; Spec 0.83; AUC 0.88^18^

**Abbreviations:** AUC = area under the ROC curve; CNN = convolutional neural network; DL = deep learning; DSC = Dice similarity coefficient; ML = machine learning; HCC = hepatocellular carcinoma; CT = computed tomography; MRI = magnetic resonance imaging; US = ultrasound.

All results reflect internal or external validation as specified; see Supplementary Table S2 for detailed study characteristics and validation status.

### 4.2 AI in liver histopathology

Automated analysis of liver histology using digital pathology and machine-learning approaches has shifted the field from semi-quantitative ordinal scoring toward continuous, reproducible measurements that can detect subtler changes in steatosis, necroinflammation, and fibrosis than traditional microscopy. A widely used quantitative metric, the collagen proportionate area (CPA), measures the percentage of biopsy occupied by collagen and correlates strongly with pathologist staging (R^2^ = 0.60–0.86 across multisite NAFLD cohorts).^21^ Similarly, qFibrosis-type workflows using second-harmonic generation imaging show strong correlation with histologic stage (r = 0.77) and discriminatory accuracy (AUC 0.87 for F ≤ 1 vs F ≥ 2; AUC 0.95 for F ≤ 3 vs F4).^22^^,^^23^

Deep-learning pipelines have extended these morphometric methods, with post-hoc analyses of phase II trials^24^^,^^25^ confirming that continuous ML-based fibrosis scores can detect antifibrotic effects missed by categorical pathologist reads, thereby offering enhanced sensitivity for clinical-trial endpoints. A major advantage of AI histopathology is its reduction in inter- and intra-observer variability. Systems such as AIM-MASH achieved κ = 1 internal reproducibility and ∼82% agreement for component grades, approximating or exceeding expert pathologists.^26^^,^^27^

Beyond reproducing conventional scores, AI-enabled histology is generating novel biomarkers predictive of outcomes. DL-based models (e.g., qFibrosis/qFIBS) identify portal-region metrics (string width/length) with AUCs >0.94 for advanced fibrosis,^28^^,^^29^ while ML-derived morphometrics correlate with noninvasive biomarkers and predict progression-free survival in advanced fibrosis cohorts.^30^^,^^31^ Composite morphologic signatures integrating steatosis proportionate area (SPA), ballooning, and inflammatory–collagen spatial metrics further improve prognostic discrimination.^32^, ^33^, ^34^

Although many studies remain single-center, recent multisite validations confirm AI–consensus reproducibility approaching human pathologist performance, reinforcing AI's augmentative clinical potential (Table 2).

**Table 2:** Selected applications of AI in liver histopathology.

Task/Metric	Sample size	AI approach	Validation	Key results
Collagen proportionate area (CPA) vs fibrosis stage	Multisite NAFLD cohorts	Automated morphometry	Multisite	R^2^ = 0.60–0.86 vs pathologist staging^21^
qFibrosis (2-photon imaging)	Validation sets	Feature-based quantitation	External	AUC 0.87 (F ≤ 1 vs F ≥ 2); AUC 0.95 (F ≤ 3 vs F4)^22^^,^^23^
Semaglutide phase II trial	*n* = 251 biopsies	Commercial ML pathology	Post-hoc	Continuous ML scores detected antifibrotic effect missed by categorical reads^24^^,^^25^
AIM-MASH reproducibility	MASH/NASH cohorts	DL-based tool	Multisite	κ = 1 internal reproducibility; ∼82% agreement; 97% fibrosis agreement^26^^,^^27^
Novel fibrosis features (portal string metrics)	Multiple cohorts	qFibrosis/qFIBS	Internal/external	AUC >0.94 for advanced fibrosis^28^^,^^29^
Prognostic biomarkers	Advanced fibrosis cohorts	DL & morphometrics	Observational	Digital histology scores predicted PFS; correlated with noninvasive biomarkers^30^^,^^31^

**Abbreviations:** AUC = area under the ROC curve; DL = deep learning; ML = machine learning; CPA = collagen proportionate area; NAFLD = nonalcoholic fatty liver disease; NASH = nonalcoholic steatohepatitis; PFS = progression-free survival.

**Note:** Most studies used internal or multisite validations; full extraction details in Supplementary Table S2.

### 4.3 AI in chronic liver diseases

AI applications in chronic liver diseases—particularly MASLD/NAFLD, NASH, viral hepatitis, and ALD—demonstrate the breadth of predictive modeling across large-scale datasets.^35^ ML models trained on national EHRs predict progression from steatosis to NASH or significant fibrosis, with AUROC = 0.79–0.87.^36^ Multicenter efforts such as LITMUS reported gradient-boosting performance of AUC 0.94 (steatosis), 0.79 (inflammation), and 0.83 (for at-risk NASH ≥ F2).^37^ Integrative algorithms combining serum, imaging, and omics outperformed conventional scores, reaching AUROC = 0.90.^38^

In viral hepatitis, ML approaches predicting DAA treatment failure achieved validation AUCs = 0.66–0.80, identifying small subsets at risk despite overall >95% success rates.^39^, ^40^, ^41^ In alcohol-related liver disease (ALD), ICU-based prognostic models trained on MIMIC-IV outperformed MELD (AUCs = 0.84–0.87 vs 0.77), highlighting ML's calibration advantage.^42^, ^43^, ^44^

Collectively, these studies (summarized in Table 3) illustrate consistent gains in discrimination over standard scores but emphasize the need for multicenter external validation to confirm generalizability.

**Table 3:** AI applications in chronic liver diseases.

Disease area	Dataset/Cohort	AI approach	Outcome	Key results
NAFLD/NASH progression	National EHR^36^	XGBoost	NASH/fibrosis progression	AUROC 0.79 (NASH), 0.87 (fibrosis)
At-risk NASH detection	LITMUS multicenter^37^	Gradient boosting	Steatosis, inflammation, fibrosis	AUCs 0.94 (steatosis), 0.79 (inflammation), 0.83 (at-risk NASH)
Noninvasive fibrosis	Biopsy-confirmed NAFLD^38^	Custom ML pipeline	≥F2 fibrosis	AUROC 0.90 (training), 0.89 (validation)
HCV treatment response	HCV-TARGET registry (*n* = 6525)^39^^,^^40^	GBM, RF, NN	DAA failure risk	AUROC = 0.66–0.69 (vs LR 0.51); sens/spec = 66%/65%
HCV treatment response	Taiwan nationwide (*n* = 34,301)^41^	XGBoost	DAA failure risk	Validation AUROC 0.803; accuracy 99.5%; sens 69.7%; spec 99.9%
ALD short-term prognosis	MIMIC-IV ICU (*n* = 2134)^42^^,^^43^	RF, XGBoost, SVM	28-day mortality	Validation AUCs 0.84–0.87 (vs MELD 0.77)

**Abbreviations:** AUC = area under the ROC curve; AUROC = area under the receiver-operating characteristic; DL = deep learning; EHR = electronic health record; GBM = gradient boosting machine; ML = machine learning; NAFLD = nonalcoholic fatty liver disease; NASH = nonalcoholic steatohepatitis; RF = random forest; SVM = support vector machine; NN = neural network; HCV = hepatitis C virus; DAA = direct-acting antiviral; ALD = alcohol-related liver disease; MELD = Model for End-Stage Liver Disease.

**Note:** Most results reflect internal or external validations; detailed data extraction available in Supplementary Table S2.

### 4.4 AI in cirrhosis and complications

**Fig. 4:**
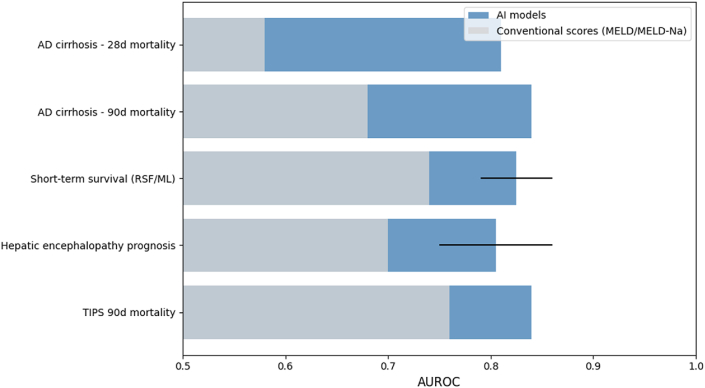
**Comparative performance of AI models versus conventional prognostic scores in cirrhosis**. AI models consistently outperformed MELD, MELD-Na, and MELD 3.0 across multiple prognostic tasks. In acute decompensated cirrhosis, an artificial neural network achieved AUCs of 0.81 (28-day) and 0.84 (90-day) versus MELD-Na (0.58, 0.68) and MELD 3.0 (0.60, 0.70). Random survival forests and other ensemble methods reached AUCs of 0.79–0.86 for short-term survival, exceeding MELD-based benchmarks. Machine-learning models for hepatic encephalopathy prognosis achieved AUCs of 0.75–0.86, again outperforming MELD and nomograms. In TIPS cohorts, incorporating imaging-derived body-composition features improved 90-day mortality prediction (AUC 0.84) over MELD alone (0.76). **Abbreviations:** AUC = area under the ROC curve; ANN = artificial neural network; CNN = convolutional neural network; DL = deep learning; ML = machine learning; RSF = random survival forest; MELD = Model for End-Stage Liver Disease; MELD-Na = MELD-sodium; TIPS = transjugular intrahepatic portosystemic shunt.

AI applications in cirrhosis and its complications have expanded rapidly, addressing the disorder's clinical and data complexity. Heterogeneous multimodal inputs and dynamic prognostic needs challenge traditional models, but modern approaches—including DL for imaging, transformer or recurrent architectures for longitudinal data, and ensemble methods (random forests, gradient boosting) for tabular clinical data—enable more accurate and individualized prediction.^45^ In a multicenter cohort of 290 patients with acute decompensated cirrhosis, an ANN achieved AUCs of 0.81 (28-day) and 0.84 (90-day) mortality, outperforming the MELD-Na score (AUCs 0.58 and 0.68) and the MELD 3.0 score (AUCs 0.60 and 0.70).^46^ Similarly, ensemble survival models such as random survival forests (RSF) reported AUCs of 0.79–0.86 for short-term prognosis, again exceeding MELD and traditional nomograms^47^^,^^48^ (Fig. 4).

AI has also demonstrated particular strength in imaging and procedural applications. DL systems for endoscopy, cross-sectional imaging, and pathology have achieved human-level accuracy. ENDOANGEL-GEV (an artificial intelligence–assisted endoscopic system designed for the automatic detection of gastroesophageal varices using deep learning algorithms), trained on >6000 endoscopic images and validated on >11,000 additional images plus a prospective cohort, achieved sensitivities >93% for esophageal and >95% for gastric varices, identifying 12.3% more intervention-requiring patients than attending endoscopists.^49^^,^^50^ Automated CT/MRI segmentation pipelines (e.g., U-Net variants) yield mean IoUs >0.75 for liver and lesion delineation, with volumetry errors <10% in surgical-planning datasets. Radiomics-based and DL predictive models report AUCs of 0.75–0.92 for hepatocellular carcinoma (HCC) detection, staging, or recurrence, frequently surpassing individual radiologist reads.^51^

The multimodal nature of cirrhosis makes it well suited for AI integration. For readmission risk, multicenter models trained on >3000 admissions improved AUCs by 4%–5% over baselines for 14- and 30-day readmissions, with consistent external calibration.^52^^,^^53^ For hepatic encephalopathy (HE), including post-TIPS HE, ML models trained on ICU/EHR datasets achieved time-dependent AUCs of 0.75–0.86 for 28-–90-day outcomes, exceeding MELD and other clinical nomograms.^54^^,^^55^ These findings illustrate AI's capacity to transform routine clinical data into early, reliable alerts for decompensation and complications, supporting triage and critical-care decisions.

AI is also transforming transplantation-related decision-making. Random survival forests, neural networks, and ensemble models have identified donor–recipient interactions and predictors of waitlist mortality beyond current allocation systems, sometimes improving graft-survival discrimination.^56^ ANN models trained on donor–recipient datasets achieved AUCs up to 0.94 for 3-month and 0.78 for 12-month graft survival in single-center validations, while simulation studies demonstrated that ANN-guided allocation altered organ utilization compared with Cox models.^57^ Integrating AI-derived imaging biomarkers (e.g., sarcopenia and body-composition metrics) into MELD frameworks also enhances risk prediction—in TIPS cohorts, inclusion of skeletal-muscle and fat-area data improved 90-day mortality prediction from AUC 0.76 (MELD) to 0.84.^58^

Collectively, these results confirm that AI models consistently outperform MELD-based benchmarks across prognosis, imaging, and transplantation tasks, while emphasizing the need for standardized external validation and interpretability for clinical adoption (Table 4).

**Table 4:** AI applications in cirrhosis and complications.

Application	Dataset/Cohort	AI approach	Comparator	Key results
Acute decompensated cirrhosis prognosis	Multicenter, *n* = 290^46^	ANN	MELD-Na, MELD 3.0	AUCs: 28 d 0.81, 90 d 0.84 vs MELD-Na 0.58/0.68
Short-term survival	Liver-failure cohorts^47^^,^^48^	RSF, ML survival models	Nomograms, MELD	AUCs 0.79–0.86 (outperforming baselines)
Variceal detection (ENDOANGEL-GEV)	>6000 training; >11,000 validation; 161 prospective^49^^,^^50^	CNN (DL)	Endoscopists	Sensitivity >93% EV, >95% GV; +12.3% vs experts
Automated segmentation/volumetry	Large CT/MRI datasets	CNN (U-Net variants)	Manual segmentation	mIoU >0.75; volumetry error <10%
HCC detection/recurrence	Radiomics + ML/DL^51^	Radiomics, CNN	Radiologists	AUCs 0.75–0.92 (often > single reader)
Readmission prediction	Multicenter (>3000 admissions)^53^	ML classifiers	Best baseline	+4%–5% AUC gain for 14- and 30-day readmissions
Hepatic encephalopathy prognosis	ICU/EHR cohorts^54^^,^^55^	RSF, ML models	MELD, nomograms	Time-dependent AUCs 0.75–0.86 (28–90 days)
Transplant graft survival	Donor–recipient datasets^56^^,^^57^	ANN, RSF, ensembles	Cox regression	AUC up to 0.94 (3 m); 0.78 (12 m); ANN allocation altered utilization
MELD + imaging features	TIPS cohort^58^	ML + body composition	MELD	90 d mortality AUC 0.84 vs MELD 0.76

**Abbreviations:** AUC = area under the ROC curve; ANN = artificial neural network; CNN = convolutional neural network; DL = deep learning; ENDOANGEL-GEV = an artificial intelligence–assisted endoscopic system designed for the automatic detection of gastroesophageal varices using deep learning algorithms. ML = machine learning; RSF = random survival forest; MELD = Model for End-Stage Liver Disease; MELD-Na = MELD-sodium; TIPS = transjugular intrahepatic portosystemic shunt; HCC = hepatocellular carcinoma.

**Validation:** unless otherwise stated, results reflect internal or multicenter external validation.

Full methodological details and dataset summaries are available in Supplementary Table S2.

### 4.5 AI in liver transplantation

Artificial intelligence and machine-learning approaches are increasingly being integrated across the liver transplantation pathway, targeting three primary domains: donor–recipient (D–R) matching, graft survival prediction, and perioperative risk modeling.^59^ Because D–R allocation involves nonlinear, high-dimensional variables, ML methods—especially neural networks and ensemble models—are well suited to capture complex interactions beyond traditional scores. Early ANN-based studies demonstrated strong short-term discrimination, achieving AUC = 0.94 for 3-month and = 0.78 for 12-month graft survival in a King's College cohort (*n* = 822), representing a ∼15% absolute improvement over MELD.^60^ Institutional validation confirmed similar accuracy: a DL model trained on 529 transplants (1058 D–R pairs) achieved 95.8% accuracy, F1 = 0.899, and AUC = 0.94, with 94.3% accuracy for in-hospital mortality.^61^ In the Korean Organ Transplantation Registry (*n* = 785), a random-forest model achieved AUCs of 0.80, 0.85, and 0.81 for 1-, 3-, and 12-month survival—outperforming MELD, BAR, and donor-risk index.^62^

Large registry studies illustrate both potential and constraints of ML. A UNOS dataset of 39,189 D–R pairs found that logistic regression (AUC 0.654) outperformed random forest (0.644), gradient boosting (0.600), and multilayer perceptron (0.599) for 5-year graft survival, reflecting the influence of data quality, imbalance, and missingness.^63^ Another UNOS analysis (*n* = 57,544) reported that a deep neural network achieved AUC 0.703 for 90-day mortality—marginally exceeding BAR (0.655) and SOFT (0.688)—indicating consistent but incremental gains.^64^ Systematic reviews agree that while ML often outperforms conventional indices for short-term outcomes, results vary with dataset characteristics, modeling strategy, and endpoint definition.^65^, ^66^, ^67^

Prediction of graft survival and postoperative complications remains the most extensively studied domain. Institutional datasets report short-term graft-survival AUCs ranging from approximately 0.78 to 0.94, whereas the performance of longer-term models (≥12 months) decreases to around 0.70–0.80 as postoperative and center-specific factors begin to influence outcomes.^57^^,^^67^ Integrating imaging-derived features into MELD frameworks enhances performance; for example, in a transplant-related TIPS cohort, adding CT-based body-composition features improved 90-day mortality prediction from AUC 0.76 to 0.84.^58^ Perioperative prediction models also perform strongly: a random-forest classifier achieved AUC 0.82 for 30-day graft failure, although accuracy decreased on external validation.^58^^,^^68^

For post-transplant acute kidney injury (AKI), a cohort of 1211 patients (incidence 30.1%) showed gradient boosting achieved AUROC 0.90 (95% CI 0.86–0.93)—far exceeding logistic regression (0.61)—with random forest performing similarly.^69^ An explainable GBM model (*n* = 894) validated robust AKI prediction while using SHAP values for interpretability.^70^ Other complication-specific models (e.g., ARDS, bleeding, early graft dysfunction) achieved AUROCs 0.72–0.88 depending on feature richness and prevalence.^70^^,^^71^ Operational and perioperative applications highlight tangible clinical benefits. In living-donor transplantation, ML-based graft-weight estimation reduced mean absolute error to 50 ± 62 g (∼10.3%), with 62% of predictions within 10% error, outperforming conventional volumetry.^72^ Similarly, multicenter ML models for massive-transfusion prediction achieved AUROCs >0.80, enabling more reliable perioperative planning.^73^

Across D–R matching, graft survival, and perioperative management, AI consistently improves short-term predictive accuracy (AUC = 0.75–0.95) in curated datasets but demonstrates performance drops on external validation. Large, high-quality registry data remain essential to mitigate overfitting and ensure generalizability, as noisy or incomplete variables can erode ML's advantage, occasionally leaving logistic regression competitive.^74^ The largest gains arise from enriched feature engineering—such as imaging or donor-quality metrics—but these may constrain generalizability. Recent work increasingly focuses on explainability (SHAP, transformer models) and fairness to promote transparent and equitable adoption in clinical transplantation^70^^,^^75^ (Table 5).

**Table 5:** AI applications in liver transplantation.

Application	Dataset/Cohort	AI approach	Comparator	Key results
D–R matching (short-term)	Spain/UK (*n* = 822)^60^	ANN	MELD	AUC 0.94 (3 m), 0.78 (12 m); +15% vs MELD at 3 m
D–R model (institutional)	529 transplants/1058 pairs^61^	DL	MELD	Accuracy 95.8%; F1 = 0.899; AUC = 0.94
D–R model (registry)	Korean registry, *n* = 785^62^	Random forest	MELD, bar, DRI	AUCs 0.80 (1 m), 0.85 (3 m), 0.81 (12 m)
Graft survival (registry)	UNOS, *n* = 39,189^63^	RF, GBM, MLP	Logistic regression	Logistic regression AUC 0.654 > ML (0.600–0.644)
Mortality prediction (registry)	UNOS, *n* = 57,544^64^	DNN	Bar, SOFT	AUC 0.703 vs BAR 0.655, SOFT 0.688
Short-term graft survival	Institutional datasets^57^^,^^67^	ANN, RF, DL	MELD	AUC 0.78–0.94 (≤3 m); 0.70–0.80 (≥12 m)
MELD + imaging features	TIPS cohort^58^	ML + CT body composition	MELD	90 d AUC 0.84 vs 0.76
Perioperative graft failure	Institutional^58^^,^^68^	RF	Clinical scores	AUC 0.82 (30 d), lower external
Post-LT AKI	*n* = 1211^69^	GBM, RF	Logistic regression	AUROC 0.90 vs 0.61
Post-LT AKI (explainable)	*n* = 894^70^	GBM + SHAP	–	Strong AUROC; interpretable predictors
Other complications (ARDS, bleeding, EGD)	Various^70^^,^^71^	ML models	Clinical models	AUROC 0.72–0.88
Graft-weight estimation	872 donors^72^	ML volumetry	Conventional volumetrics	MAE 50 g (10.3% error); 62% within 10%
Massive transfusion prediction	Multicenter^73^	ML classifiers	Clinical heuristics	AUROC >0.80

**Abbreviations:** AUC = area under the ROC curve; ANN = artificial neural network; BAR = Balance of Risk score; DL = deep learning; DNN = deep neural network; DRI = donor risk index; GBM = gradient boosting machine; MAE = mean absolute error; ML = machine learning; MLP = multilayer perceptron; RF = random forest; SHAP = Shapley Additive Explanation; SOFT = Survival Outcomes Following Transplantation; TIPS = transjugular intrahepatic portosystemic shunt.

**Validation:** Unless stated, results reflect internal or multicenter external validation. Full methodological and dataset details are provided in Supplementary Table S2.

### 4.6 AI in hepatology clinical practice

AI and ML models are increasingly leveraging structured and unstructured electronic health record (EHR) data to enhance risk stratification, prognostication, and clinical workflow in hepatology. In a primary-care cohort of 344 patients (56.4% women), a random-forest model predicting hepatic fibrosis (liver stiffness >7 kPa) using routine variables (age, sex, diabetes, hypertension, BMI, FIB-4, and lipids) achieved AUC 0.75 (95% CI 0.67–0.84), NPV 91.5%, and PPV 40.0%.^76^ At standard thresholds (BMI ≥30, FIB-4 ≥ 1.45), discrimination decreased to AUC = 0.65, with PPV = 50% and unchanged NPV = 91%.^76^^,^^77^

In a multicenter Taiwanese cohort of 5878 cirrhotic patients, an XGBoost model achieved AUC 0.86 (95% CI 0.83–0.88) for early hepatic-encephalopathy (HE) prediction, outperforming neural networks (0.79), support-vector machines (0.77), and MELD (0.74). At a probability threshold 0.25, sensitivity 72%, specificity 80%, PPV 48%, and NPV 92% were achieved.^78^ Together, EHR-based ML models achieve moderate-to-high discrimination (AUC = 0.75–0.86) and consistently high NPV, supporting their utility for population-level screening and early complication detection.

NLP extends AI's reach by mining unstructured text such as notes and radiology reports. In the Mount Sinai BioMe program (*n* = 38,575), NLP applied to clinical notes identified 2281 NAFLD cases, outperforming ICD codes (sensitivity 0.93 vs 0.28) and free-text search (0.81), with F_2_ score 0.92 vs 0.34 (ICD) and 0.81 (text search).^79^^,^^80^ Among detected cases, 29.5% progressed to NASH or cirrhosis; notably, NAFLD mentioned only in radiology reports but not in notes often later progressed, with a median ∼1057 days (∼2.9 years) to clinical recognition. Another validation study on 800 radiology reports for steatotic liver disease (SLD) achieved sensitivity 100%, PPV 88.5%, and NPV 100%, with application to the full imaging cohort flagging ∼72.2% as SLD.^81^ However, a systematic review of 53 NLP studies in gastroenterology/hepatology found only 13 (24.5%) addressed liver disease, of which = 25% had low risk of validation bias and = 9.4% reported explainability or open-source code—underscoring gaps in transparency and generalizability.^82^

Decision-support systems (DSS) and AI-based clinical decision support (AI-CDS) remain underexplored but conceptually promising. A multicenter qualitative analysis of 53 liver-transplant providers highlighted essential design principles for trustworthy AI-CDS: transparency in model development, interpretability, bias mitigation, and contextual use as a clinician aid rather than replacement.^83^ Collectively, these findings show that structured-data ML, NLP, and early DSS applications can enhance identification, risk prediction, and workflow efficiency, while safe implementation depends on external validation, interpretability, and clinician trust (Table 6).

**Table 6:** AI applications in hepatology clinical practice.

Application	Dataset/Cohort	AI approach	Comparator	Key results
Fibrosis prediction (EHR)	Primary care, *n* = 344^76^^,^^77^	Random forest	Standard thresholds (BMI, FIB-4)	AUC 0.75 (NPV 91.5%, PPV 40%); thresholds AUC = 0.65
Early HE prediction	Taiwan *n* = 5878 cirrhotics^78^	XGBoost	NN, SVM, MELD	AUC 0.86 vs NN 0.79, SVM 0.77, MELD 0.74; Sens 72%, Spec 80%, NPV 92%
NAFLD case identification	Mount Sinai BioMe, *n* = 38,575 [79, 80]	NLP (clinical notes)	ICD codes, text search	Sens 0.93 vs ICD 0.28; F_2_ 0.92 vs 0.34; 29.5% progressed to NASH/cirrhosis
SLD detection (radiology)	*n* = 800 validated; ∼26,700 full cohort^81^	NLP	Manual review	Sens 100%, PPV 88.5%, NPV 100%; 72.2% flagged as SLD
NLP systematic review	53 studies^82^	–	–	24.5% liver-focused; ∼25% low bias; ∼9.4% shared code/explainability
Decision support (transplant listing)	53 providers, multicenter^83^	Qualitative CDS study	–	Themes: Transparency, interpretability, bias mitigation, contextual use

**Abbreviations:** AI = artificial intelligence; AUC = area under the ROC curve; CDS = clinical decision support; DL = deep learning; EHR = electronic health record; HE = hepatic encephalopathy; ML = machine learning; NAFLD = nonalcoholic fatty liver disease; NASH = nonalcoholic steatohepatitis; NPV = negative predictive value; NN = neural network; PPV = positive predictive value; SVM = support-vector machine; SLD = steatotic liver disease.

**Validation:** Unless specified, performance refers to internal or multicenter external validation. Full dataset and methodological details appear in Supplementary Table S2.

## 5 Discussion

### 5.1 Challenges and limitations

**Fig. 5:**
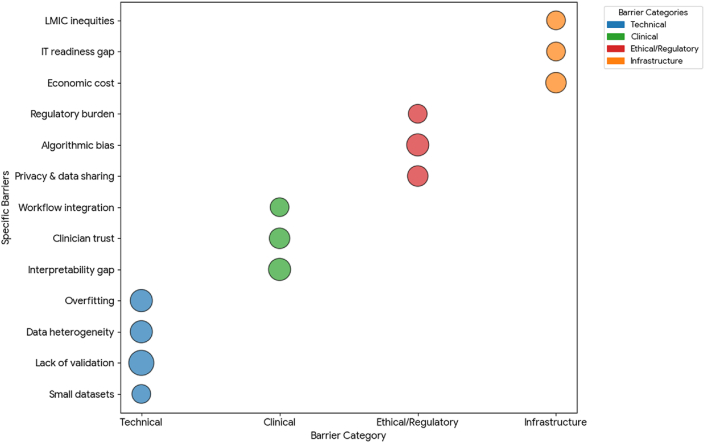
**Challenges and limitations of AI in hepatology.** Recurring barriers were synthesized into four categories: **technical** (blue – small datasets, limited external validation, data heterogeneity, overfitting); clinical (green – low interpretability, limited clinician trust, workflow integration); ethical/regulatory (red – privacy, data-sharing restrictions, algorithmic bias, compliance burden); and infrastructural/economic (orange – implementation cost, IT readiness gaps, LMIC inequities). Bubble size denotes the relative frequency of each barrier. Collectively, these highlight that beyond accuracy, robust validation, explainability, fairness, and regulatory readiness are essential for safe clinical translation. **Abbreviations:** AI = artificial intelligence; AUC = area under the ROC curve; DL = deep learning; EHR = electronic health record; GDPR = General Data Protection Regulation; HIPAA = Health Insurance Portability and Accountability Act; LMIC = low- and middle-income countries; ML = machine learning.

AI in hepatology continues to face several interrelated technical, clinical, ethical, and infrastructural barriers that constrain generalizability and translation (Fig. 5). A primary limitation is data heterogeneity—differences in imaging protocols, scanner vendors, staining techniques, EHR codification, and laboratory assays—which cause distribution shifts that degrade model performance across sites. Empirical evaluations confirm this: of 21 diagnostic-model abstracts recommending clinical use, 95.2% lacked external validation.^84^ Broader reviews likewise show low external-validation rates; only a minority of published models undergo independent testing, and few are prospectively evaluated.^85^^,^^86^ When external validation occurs, AUROC frequently declines by 0.05–0.20, indicating overfitting and dataset-specific tuning. Small single-center cohorts (*n* = 200–1200) further limit reproducibility, whereas registry-scale datasets (>10,000 records) improve statistical power but often lack detailed imaging or pathology data.^87^^,^^88^

Clinical and workflow barriers remain equally significant. Integrating AI into hepatology practice is a socio-technical challenge: clinicians cite alert fatigue, workflow interruptions, and unclear responsibility for algorithmic actions. Reviews of real-world deployments identify interpretability and clinician trust as leading obstacles.^89^ Across pilots, only 40%–60% of clinicians expressed confidence in opaque AI outputs, and adoption rates for non-explainable alerts averaged below 30%.^90^^,^^91^ In contrast, decision-support tools incorporating explainability features (e.g., SHAP, LIME, attention maps) improved acceptance and reduced adjudication times, though randomized evidence of patient-level benefit is still lacking.^55^^,^^70^ These findings emphasize that technical accuracy alone is insufficient—clinician usability and transparency are prerequisites for adoption.

Ethical and regulatory concerns further constrain implementation. Reviews of ethical barriers show frequent mention of privacy, bias, and accountability.^92^ Under GDPR and HIPAA, data-sharing timelines often extend by months, requiring pseudonymization and formal agreements, which in multi-institutional European projects forced a shift toward federated or synthetic-data approaches.^93^ Algorithmic bias is quantifiable: subgroup analyses document 5%–15% performance gaps between demographic groups, with sensitivity sometimes dropping from 92% to 78% in under-represented populations.^92^^,^^94^ Recent regulatory guidance now mandates fairness auditing and subgroup performance reporting before approval.^95^^,^^96^

Economic and infrastructural limitations disproportionately affect low- and middle-income countries (LMICs). Global surveys reveal AI readiness correlates with health-IT investment: hospitals spending > US$5000 per bed on IT were three times more likely to pilot AI tools than those spending < US$1000.^89^^,^^97^ Resource disparities limit availability of digitized imaging and pathology data, preventing model reuse without domain adaptation. Estimated deployment costs remain substantial—US$50 k–200 k per scanner for digital pathology hardware, plus recurring cloud or on-premise compute and engineering staff.^93^^,^^94^ These inequities risk widening the implementation gap between high- and low-resource centers, raising questions of fairness in hepatology AI access.

This scoping review is limited by (1) restriction to English-language, peer-reviewed studies (preprints excluded unless published); (2) possible omission of older studies using non-AI terminology, which was mitigated by retrospective inclusion of synonyms such as “automated analysis” and “computer-assisted diagnosis” (Supplementary Table S1); and (3) heterogeneity in outcome definitions and metrics, which precluded quantitative meta-analysis. These limitations were addressed through expanded search terms, dual screening, and transparent data-charting procedures.

### 5.2 Future directions

AI research in hepatology is shifting toward multimodal, explainable, and privacy-preserving designs that align with clinical complexity and regulatory expectations. Multimodal AI, integrating imaging, pathology, clinical data, and molecular omics, directly addresses liver disease heterogeneity and the multimodal nature of care.^98^ Recent reviews highlight a surge of such studies, with fused architectures (transformers, graph neural networks, and early/late fusion models) outperforming unimodal approaches and capturing cross-domain biological signals.^99^ In liver applications, MRI-radiomics plus laboratory or genomic data improved internal AUCs by 0.03–0.12 for microvascular invasion and recurrence prediction.^98^^,^^100^ This reflects a clear clinical rationale—morphologic, biochemical, and molecular layers jointly enable finer stratification.^101^^,^^102^

Explainable AI (XAI) has become a practical necessity rather than a secondary feature. Methods such as SHAP, attention maps, and case-based reasoning enhance transparency and clinician trust.^103^ In a large NHANES cohort (*n* = 5281), an XGBoost + SHAP model identified top biochemical and anthropometric predictors of high-risk MASH (AUC 0.95), illustrating how interpretability supports adoption.^104^ Clinician-evaluation studies confirm that transparency markedly increases acceptance, emphasizing that explainability must be embedded in model design.^105^^,^^106^ Federated learning (FL) offers a viable path for data-secure collaboration. Simulations for ultrasound-based steatosis detection showed FL achieving AUC 0.93—comparable to centralized training (0.90) and well above single-center models (0.83).^107^ These privacy-preserving frameworks satisfy GDPR and HIPAA constraints and could underpin future international, multicenter hepatology AI trials.^108^

AI-driven precision medicine is emerging through integrative models that identify molecular and imaging-based subphenotypes with prognostic and therapeutic relevance. Multimodal NASH classifiers achieved AUC >0.80 for treatment-response prediction, supporting use in trial enrichment and personalized therapy.^109^^,^^110^ Wearables and digital biomarkers are extending hepatology AI into home monitoring. Longitudinal wearable data discriminated covert hepatic encephalopathy with AUC 0.79, while combined physiologic and biochemical signals achieved sensitivities of 70%–90% for detecting early decompensation.^52^^,^^111^^,^^112^

Priority recommendations for future research:

(1) Conduct large-scale, prospective, multicenter validation trials to ensure generalizability.
(2) Develop multimodal and explainable architectures integrating EHR, imaging, pathology, omics, and wearable data.
(3) Adopt federated and privacy-preserving learning frameworks to enable secure cross-institutional collaboration.
(4) Implement standardized reporting (TRIPOD-AI, CONSORT-AI) and fairness auditing across demographic and geographic subgroups.
(5) Focus on real-world workflow integration and clinician usability to translate algorithmic accuracy into patient benefit.

Collectively, these directions chart a path from promising prototypes toward equitable, explainable, and validated AI systems ready for clinical hepatology.

### 5.3 Knowledge gaps identified

Despite rapid progress, key evidence gaps persist across pediatric, rare, and underrepresented populations.

In pediatric hepatology, machine-learning models show promise for NAFLD and NASH detection, yet validation remains sparse and sample sizes small.^113^ Most AI studies continue to focus on adult disease, leaving pediatric applications underrepresented.^85^^,^^86^ For rare liver diseases, early algorithms (e.g., for acute hepatic porphyria) demonstrate feasibility but struggle with generalizability due to limited, fragmented datasets.^114^ Broader reviews confirm that small, heterogeneous cohorts are the dominant translational barrier.

Longitudinal and real-world validation remain scarce. Many reports rely on retrospective, single-center data; performance often degrades when externally tested.^84^^,^^115^ Prospective multicenter studies with standardized outcomes are urgently needed. Population diversity is another major gap. Models trained on homogeneous datasets exhibit performance drops across sex, ethnicity, and region, reinforcing calls for inclusive training data.^116^ Women, minorities, and LMIC populations remain underrepresented, raising fairness and regulatory challenges. Policy and reporting standards are inconsistently applied: few studies follow CONSORT-AI, SPIRIT-AI, or TRIPOD-AI, and lack of harmonized data-sharing frameworks limits federated validation.^85^ Regulatory agencies (FDA, EMA) have yet to issue hepatology-specific AI guidance, slowing translation.

In summary, bridging these gaps requires:

(1) Diverse, high-quality datasets spanning demographics and geographies.
(2) Prospective multicenter and longitudinal validation to assess durability.
(3) Adherence to standardized reporting and transparency guidelines.
(4) Policies incentivizing equitable data sharing and inclusion of LMIC centers.

Aligning hepatology AI research with these principles will accelerate movement from feasibility studies to clinically deployable, trustworthy tools.

## 6 Conclusion

Artificial intelligence is increasingly transforming hepatology, spanning imaging, pathology, chronic liver disease management, cirrhosis, and transplantation. These developments demonstrate tangible gains in diagnostic accuracy, prognostic modeling, and workflow support, underscoring AI's capacity to augment—not replace—clinical expertise. However, translation into everyday practice remains limited by inconsistent external validation, interpretability gaps, regulatory ambiguity, and inequitable data representation across regions. To realize its full potential, future research must prioritize prospective multicenter validation, integration of multimodal and real-world data, transparent and fair model design, and strict adherence to TRIPOD-AI and CONSORT-AI standards. With these foundations, AI can evolve from experimental promise to an equitable and reliable component of global hepatology care.

## CRediT authorship contribution statement

**Kirolos Eskandar:** Writing – review & editing, Writing – original draft, Visualization, Validation, Supervision, Software, Resources, Project administration, Methodology, Investigation, Formal analysis, Data curation, Conceptualization.

## Informed consent

Not Applicable.

## Ethics statement

Not Applicable.

## Data availability statement

All data extracted or analyzed in this review are included within the manuscript and Supplementary Materials.

## Declaration of Generative AI and AI-assisted technologies in the writing process

No Artificial Intelligence tools have been used.

## Funding

This research received no external funding.

## Declaration of competing interest

The authors declare no conflict of interest.
