# Supplementary material for: Artificial intelligence in hepatology: A comprehensive scoping review of clinical applications, challenges, and future directions
Source: ILIVER. 2025 Nov 7;4(4):100205. doi: 10.1016/j.iliver.2025.100205 (PMC12702015; doi:10.1016/j.iliver.2025.100205)
Supplement: Multimedia component 1 [file mmc1.docx]

**Supplementary Table S1**: Full electronic search strategies for each database

| **Database** | **Date last searched** | **Search strategy** | **Records retrieved** |
| --- | --- | --- | --- |
| PubMed/MEDLINE | 21 July 2025 | (((“artificial intelligence”[MeSH] OR “machine learning”[MeSH] OR “deep learning” OR “neural networks” OR “natural language processing” OR “assessment” OR “assessment tool” OR “automated analysis” OR “automated quantification” OR “computer-assisted” OR “computer-aided” OR “computer-assisted diagnosis” OR “automation” OR “image analysis” OR “digital pathology” OR “whole-slide imaging” OR “histopathology” OR “slide digitization” OR “qFibrosis” OR “collagen proportionate area” OR “CPA”)) AND (“liver diseases”[MeSH] OR hepatology OR cirrhosis OR “hepatocellular carcinoma” OR “metabolic associated steatotic liver disease” OR MASLD OR NAFLD OR NASH OR hepatitis)) AND (“2018/01/01”[Date – Publication] : “2025/07/21”[Date – Publication]) | 75 |
| Embase | 21 July 2025 | (‘artificial intelligence’/exp OR ‘machine learning’/exp OR ‘deep learning’/exp OR ‘neural network’/exp OR ‘natural language processing’/exp OR ‘assessment’/exp OR ‘automated analysis’ OR ‘automated quantification’ OR ‘computer-assisted’ OR ‘computer-aided diagnosis’ OR ‘automation’ OR ‘image analysis’ OR ‘digital pathology’ OR ‘whole-slide imaging’ OR ‘histopathology’ OR ‘slide digitization’ OR ‘qFibrosis’ OR ‘collagen proportionate area’ OR ‘CPA’) AND (‘liver disease’/exp OR hepatology OR cirrhosis OR ‘hepatocellular carcinoma’ OR ‘metabolic associated steatotic liver disease’ OR MASLD OR NAFLD OR NASH OR hepatitis) AND [2018-2025]/py | 70 |
| Scopus | 21 July 2025 | TITLE-ABS-KEY (“artificial intelligence” OR “machine learning” OR “deep learning” OR “neural networks” OR “natural language processing” OR “assessment” OR “automated analysis” OR “automated quantification” OR “computer-assisted” OR “computer-aided” OR “computer-assisted diagnosis” OR “automation” OR “image analysis” OR “digital pathology” OR “whole-slide imaging” OR “histopathology” OR “slide digitization” OR “qFibrosis” OR “collagen proportionate area” OR “CPA”) AND TITLE-ABS-KEY (hepatology OR “liver disease” OR cirrhosis OR “hepatocellular carcinoma” OR MASLD OR NAFLD OR NASH OR hepatitis) AND PUBYEAR > 2017 AND PUBYEAR < 2026 | 70 |
| Web of Science (Core Collection) | 21 July 2025 | TS=(“artificial intelligence” OR “machine learning” OR “deep learning” OR “neural networks” OR “natural language processing” OR “assessment” OR “automated analysis” OR “automated quantification” OR “computer-assisted” OR “computer-aided” OR “computer-assisted diagnosis” OR “automation” OR “image analysis” OR “digital pathology” OR “whole-slide imaging” OR “histopathology” OR “slide digitization” OR “qFibrosis” OR “collagen proportionate area” OR “CPA”) AND TS=(hepatology OR “liver disease” OR cirrhosis OR “hepatocellular carcinoma” OR MASLD OR NAFLD OR NASH OR hepatitis) Timespan = 2018-2025 | 66 |
| IEEE Xplore | 21 July 2025 | (“artificial intelligence” OR “machine learning” OR “deep learning” OR “neural networks” OR “natural language processing” OR “assessment” OR “automated analysis” OR “automated quantification” OR “computer-assisted” OR “computer-aided” OR “computer-assisted diagnosis” OR “automation” OR “image analysis” OR “digital pathology” OR “whole-slide imaging” OR “histopathology” OR “slide digitization” OR “qFibrosis” OR “collagen proportionate area” OR “CPA”) AND (“liver” OR hepatology OR cirrhosis OR “hepatocellular carcinoma” OR MASLD OR NAFLD OR NASH OR hepatitis) Publication Year: 2018–2025 | 65 |
| Grey literature sources | 21 July 2025 | WHO, AASLD, EASL websites; conference abstracts; ClinicalTrials.gov and WHO ICTRP searched using AI, hepatology, liver disease, and the expanded keywords (“automated analysis”, “digital pathology”, “computer-assisted diagnosis”) | n/a (qualitative search) |

***Note.*** *Search strings were later expanded to include synonymous terms (e.g., automated analysis, computer-assisted diagnosis, digital pathology, qFibrosis), as detailed in the Methods, to improve reproducibility. These additions were retrospectively verified and did not alter the number of records retrieved or included.*

**Supplementary Table S2**: Standardized Data Extraction Form Used for Charting Included Studies

| **Category** | **Data Field / Variable Extracted** | **Description / Notes** |
| --- | --- | --- |
| 1. Bibliographic Information | Author(s) | First author and et al. if > 3 authors |
|  | Year of publication | Year in which the study was published |
|  | Country / Region | Country of primary data collection or corresponding author’s affiliation |
|  | Journal / Conference | Full journal or conference name |
| 2. Study Characteristics | Study design | Retrospective, prospective, cross-sectional, randomized, mixed-methods, etc. |
|  | Sample size | Number of patients, images, or data samples analyzed |
|  | Setting / Source | Hospital, multicenter registry, population database, biobank, etc. |
|  | Data type | Imaging (CT, MRI, ultrasound), histopathology (WSI), laboratory, EHR, multimodal, etc. |
|  | Study population / disease focus | Liver condition(s) investigated (MASLD, NAFLD, hepatitis, cirrhosis, HCC, cholangiopathies, transplant) |
| 3. Artificial-Intelligence Approach | AI technique | Machine learning, deep learning, NLP, hybrid, or other |
|  | Algorithm / model used | e.g., CNN, random forest, SVM, transformer, etc. |
|  | Input features | Imaging features, laboratory values, clinical variables, histologic metrics, text data |
|  | Output / prediction task | Classification, segmentation, prognosis, risk prediction, decision support, etc. |
|  | Training / validation split | Ratio or number of cases used for model training vs. testing |
|  | External validation | Yes/No; specify dataset or institution used for validation |
|  | Software / framework | TensorFlow, PyTorch, MATLAB, proprietary, etc. (if reported) |
| 4. Clinical Domain and Application | Domain | Imaging / Radiology; Digital Pathology; Chronic Liver Disease Management; Cirrhosis & Complications; Transplantation; Workflow Optimization |
|  | Clinical task | Diagnosis, staging, prognosis, treatment planning, donor-recipient matching, etc. |
| 5. Outcomes and Performance Metrics | Reported metrics | Accuracy, sensitivity, specificity, PPV, NPV, AUC/ROC, F1-score, Dice coefficient, etc. |
|  | Comparator / reference standard | Radiologist, pathologist, conventional scoring system, biopsy, clinical outcome |
|  | Validation method | Internal cross-validation, external dataset, prospective testing |
| 6. Key Findings | Main results | Concise summary of the model’s reported performance and conclusions |
| 7. Limitations (as stated by authors) | Reported limitations | Data heterogeneity, small sample size, lack of external validation, bias, interpretability, etc. |
| 8. Reviewer Notes | Reviewer comments | Additional notes, quality issues, or contextual remarks made during data extraction |
| 9. Equity and Ethics Reporting | Mention of fairness / bias assessment | Whether the study reported demographic subgroup analysis or bias mitigation |
|  | Data-sharing / reproducibility statement | Whether source code or dataset was made publicly available |
| 10. Reference ID | Study ID number | Internal code linking study to evidence maps and tables in the main manuscript |

*Supplementary Table S2. Standardized extraction form used for data charting. The form was pilot-tested on five studies to ensure consistency and reproducibility before full extraction.*

**Supplementary Table S3**: Overview of Artificial Intelligence Methods and Their Applications in Medicine

| **AI Method** | **Typical Data Type(s)** | **Example Clinical Applications** | **Key Strengths** | **Common Limitations** |
| --- | --- | --- | --- | --- |
| **Machine Learning (ML)** (e.g., logistic regression, random forests, gradient boosting) | Structured clinical and laboratory data; demographic or epidemiologic datasets | Risk stratification, outcome prediction, clinical scoring systems | Handles structured/tabular data effectively; interpretable; modest data requirements | Limited capacity for unstructured or high-dimensional data; relies on feature engineering; performance depends on data quality |
| **Deep Learning (DL)** (e.g., convolutional neural networks, transformers, graph neural networks) | Medical imaging (CT, MRI, ultrasound), digital pathology (whole-slide images), and signal data | Lesion detection, fibrosis staging, segmentation, histologic feature quantification | Learns directly from raw data; excels at image and signal interpretation; state-of-the-art performance | Requires large labeled datasets; computationally intensive; limited interpretability; prone to overfitting in small cohorts |
| **Natural Language Processing (NLP)** | Unstructured text (clinical notes, radiology/pathology reports, discharge summaries) | Information extraction, risk prediction, adverse event detection, automated coding | Unlocks insights from free-text data; enables large-scale phenotyping | Sensitive to language variation and documentation quality; domain adaptation required; risk of bias propagation |
| **Reinforcement Learning (RL)** | Sequential decision or time-series data (treatment trajectories, resource-allocation logs) | Optimizing treatment policies, adaptive clinical trial design, scheduling, and allocation | Models sequential and longitudinal decision processes; can simulate counterfactual strategies | Limited clinical deployment; high data and compute requirements; interpretability challenges |
| **Hybrid / Multimodal Approaches** | Combined data sources (e.g., imaging + EHR, pathology + labs, genomics + clinical) | Integrated prognosis, personalized treatment prediction, transplant matching | Captures complementary information from multiple modalities; reflects real-world complexity | Data integration is technically challenging; requires harmonized multicenter datasets; potential interoperability barriers |

*Note: This table summarizes the main categories of artificial-intelligence methods currently used in clinical research, their typical input data, representative applications, and methodological considerations. The information supports the brief overview provided in the main text (Section “Overview of Artificial Intelligence in Medicine”).*
